# Nanoparticles synthesis in microwave plasmas: peculiarities and comprehensive insight

**DOI:** 10.1038/s41598-023-49818-3

**Published:** 2024-02-26

**Authors:** Karim Ouaras, Guillaume Lombardi, Khaled Hassouni

**Affiliations:** 1 LSPM, CNRS, Université Paris 13 Sorbonne Paris Cité, 99 Av. J. B. Clément, 93430 Villetaneuse, France; 2 LPICM, CNRS, Ecole Polytechnique, Institut Polytechnique de Paris, 91128 Palaiseau, France

**Keywords:** Nanoparticles, Plasma physics

## Abstract

Low-pressure plasma processes are routinely used to grow, functionalize or etch materials, and thanks to some of its unique attributes, plasma has become a major player for some applications such as microelectronics. Plasma processes are however still at a research level when it comes to the synthesis and functionalization of nanoparticles. Yet plasma processes can offer a particularly suitable solution to produce nanoparticles having very peculiar features since they enable to: (i) reach particle with a variety of chemical compositions, (ii) tune the size and density of the particle cloud by acting on the transport dynamics of neutral or charged particles through a convenient setting of the thermal gradients or the electric field topology in the reactor chamber and (iii) manipulate nanoparticles and deposit them directly onto a substrate, or codeposit them along with a continuous film to produce nanocomposites or (iv) use them as a template to produce 1D materials. In this article, we present an experimental investigation of nanoparticles synthesis and dynamics in low-pressure microwave plasmas by combining time-resolved and in-situ laser extinction and scattering diagnostics, QCL absorption spectroscopy, mass spectrometry, optical emission spectroscopy and SEM along with a particle transport model. We showed for the first time the thermophoresis-driven dynamic of particle cloud in electrodless microwave plasmas. We showed that this effect is linked to particular fluctuations in the plasma composition and results in the formation of a void region in the bulk of the plasma surrounded by a particle cloud in the peripherical post-discharge. We also reveals and analyze the kinetics of precursor dissociation and molecular growth that result in the observed nanoparticle nucleation.

## Introduction

Dusty or complex plasmas studies are of great importance in the context of key-technological areas such as energy and environment^1–4^, microelectronics^5–8^, fusion devices^9–16^, thin film deposition^17–20^, nanotechnology and nano-objects^21–26^, sensors^27^, catalyst^28^, biomedical applications^29–32^. These plasmas are also investigated for understanding fundamental aspects in several fields such as astrophysics or complex system dynamics^33–43^. In these topics, a number of studies have been carried out to describe the processes leading to the formation and transport of nano- and micro-particles in discharge plasmas. For some industrial applications, nanoparticles are useful for the improvement of nanomaterial-based devices^44^. Nanoparticle (NP) populations need however to be finely tuned in terms of size distribution and number density in order to meet industrial demand. On the other hand, NPs formation can represent a serious limitation for physical/chemical vapor deposition processes used for thin film synthesis. In these processes, detrimental solid particles may form due to molecular growth and homogeneous nucleation in the gas phase. Their presence in the plasma may lead to the contamination of the deposited material, thus strongly affecting its desirable functionalities^45^. It may also lead to safety issues for example in fusion plasma devices where NPs produced by the interaction between the plasma and its facing components can take up deuterium and cause radioactive emission in case of air ingress and explosions^46,47^. Considering their high toxicity, NPs can also generate health problems for human operators.

The understanding of NPs formation process along with the basic phenomena induced by the presence of such particles in the plasma phase can be achieved using laboratory discharge plasmas. The dusty plasma systems commonly used in the laboratory are capacitively coupled radio frequency (CCRF) glow discharges that have been thoroughly investigated. As a matter of fact, a large number of studies have been undertaken in such discharges where solid particles were produced using a variety of molecular precursors in C/H and Si/H systems, *e. g.,* SiH_4_, CH_4_, C_2_H_2_, etc., or through sputtering of metal or graphite electrodes^14,18,48–51^. These plasmas enabled understanding the mechanisms underlying not only NPs nucleation, charging and growth but also a variety of collective phenomena and/or characteristics experienced by dust clouds such as the formation of void region inside the plasma^52–54^, the existence of typical waves and instabilities^41^, and the possibility to produce macroscopic coulomb crystals that may be readily analyzed^42,55,56^. Unlike the abovementioned RF discharges, low-pressure microwave (MW) discharges are very much less studied as far as NPs formation and dynamics are concerned. Yet these discharges are increasingly used in the industry, especially for etching, deposition and surface treatment processes^57–60^ as they provide a larger plasma density and an enhanced plasma reactivity. Furthermore, the use of lower excitation frequencies in this kind of discharges, *e.g*., 915 MHz instead of classical 2.45 GHz, allows process scale up and treatment of much larger substrates^61,62^. Despite these advantages, high-density microwave discharges that use hydrocarbon gases (e.g. C_2_H_2_, CH_4_…) may be also the subject of clustering processes and solid particles formation^37,63–68^. This may affect the performance of plasma processes dedicated to material deposition, etching or surface-treatment. It is therefore necessary to understand and qualify the NPs growth kinetic and transport in such discharges. Further, investigating electrodeless microwave discharge enables exploring NPs formation over ranges of discharge conditions, i.e., pressure and power density, that are much wider than those characterizing capacitive coupled RF discharges. In particular, these systems make it possible to investigate how the plasma density, the electron temperature and the gas heating may affect both NPs formation and cloud dynamics.

In this article, we present a comprehensive study on the formation kinetics and population dynamics of NPs produced in low-pressure microwave plasmas. We focus on addressing some key-questions such as: (i) what are the mechanisms that govern the growth and agglomeration processes of NPs? (ii) how does the plasma behave in response to NPs formation in terms of electron and molecular precursors kinetic?

For this purpose, we performed this study using C_2_H_2_ as a hydrocarbon precursor as it has been used in a large number of fundamental investigations and of process development studies, e.g. a–C:H films^69,70^, diamond films^65^, graphene films^71^, cosmic dust analogs^33^, etc. The chemistry involved in NPs nucleation was investigated using Quantum Cascade Laser Absorption spectroscopy (QCLAS) coupled to Mass Spectrometry (MS). The NPs growth kinetic was assessed using laser extinction method while the dynamics of the NPs cloud was investigated using laser scattering imaging supported by a simple model that makes use of a momentum balance to evaluate the transport of NPs. In the experimental section hereafter, we present the investigated discharge setup along with the characterization techniques and the methodology used to investigate the dynamics of electrons, plasma emission, and molecular precursor densities during NPs formation on one hand, and the growth kinetics and the collective dynamics of the NPs cloud on the other hand. In the results and discussion section, we present the main outcomes of this study, starting with NPs growth kinetics and transport dynamics that clearly show the existence of multi-generational growth in the investigated discharges with a key-role of thermophoresis as far as transport phenomena and collective dynamics, especially void formation, are concerned. Then the very peculiar dynamics of electron density, discharge emission and hydrocarbon species densities during NPs formation and growth are discussed and correlated to the results obtained on the NPs-cloud dynamics. In the last section, we summarize the main results obtained in this work and propose an overall picture of the major phenomena that govern particle formation and dynamics in the considered discharge.

## Experimental

### MW Plasma reactor set-up and methodology

**Figure 1:**
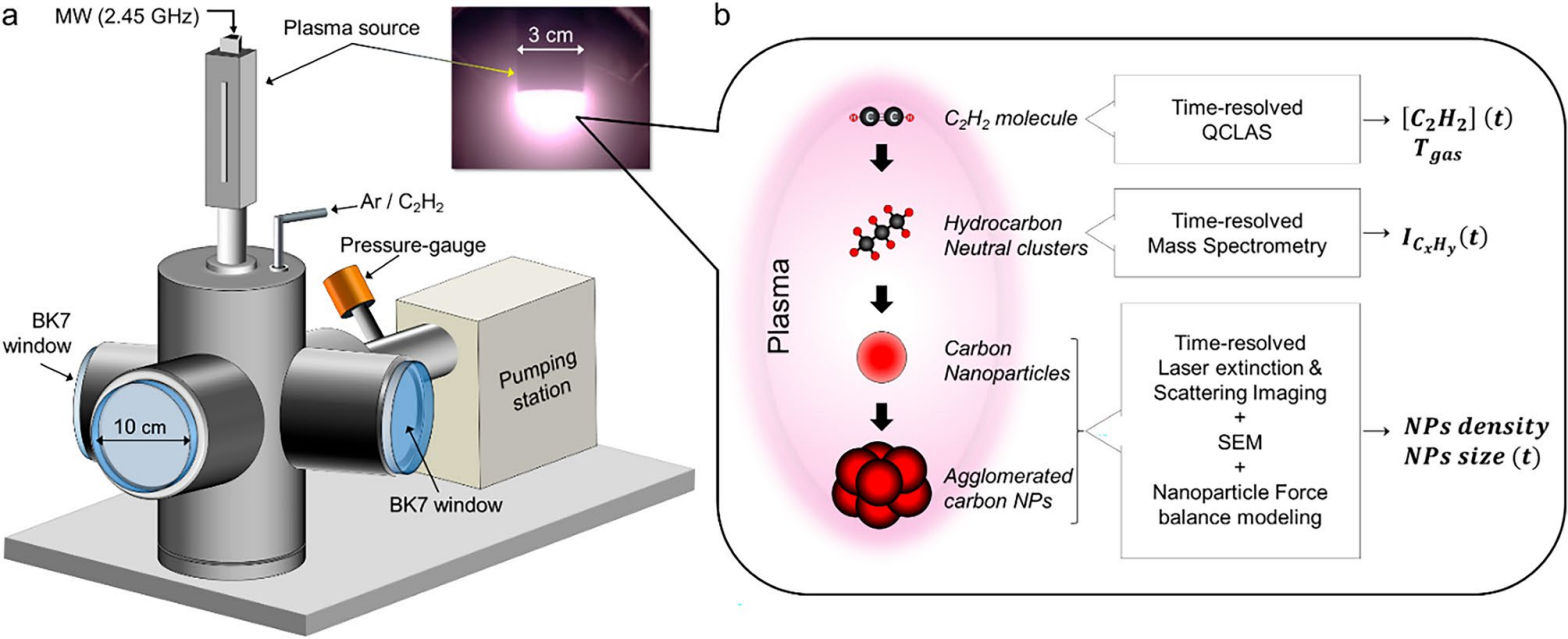
(**a**) Schematic of the microwave plasma reactor and (**b**) the multi-step methodology used for investigating the NPs formation process.

Figure 1a shows a schematic of the microwave (MW) plasma reactor. The reactor housing consists of a 6-way crosses stainless steel vacuum chamber of 5.12 L. This configuration enables performing several diagnostics in parallel so that the plasma and NPs can be probed simultaneously. The chamber is connected to a pumping plant (turbo-molecular pump/backing pump combination) that allows achieving a base pressure of about 10^−4^ Pa. The MW (*f* = 2.45 GHz) plasma source (BOREAL plasma) is mounted on the chamber through either the top or the lateral flange. The plasma source is powered by a MW generator GMP 12 KE/D (SAIREM company). A picture of the plasma is shown in Fig. 1a. Experiments were conducted under the following conditions: pressure of about 50 Pa, an equimolar Ar/C_2_H_2_ mixture and a total flowrate of 3 sccm. This set of conditions was selected as it ensures both an optimal plasma power coupling and efficient NPs production. Under such conditions, the plasma exhibits a mean electron density *n*_*e*_ and an electron temperature *T*_*e*_ of the order of 10^16^ m^−3^ and 2 eV, respectively. The experimental study was carried out according to the multi-step approach schematized in Fig. 1b. Each step of this approach corresponds to a given stage in the particle formation process, i.e., active precursor formation, molecular growth, and nucleation and particle growth. Each of these stages was characterized by analyzing the plasma phase and/or the NPs kinetic/dynamics using time-resolved, in situ and/or ex-situ diagnostics. The experimental results were compared to those obtained from a 2D NP’s-momentum balance model, which enabled interpreting the experimental measurements and shedding light on the major phenomena and processes involved in NPs nucleation, growth and dynamics in the investigated microwave discharge. All the diagnostics introduced in Fig. 1b are described in details hereafter.

### In-situ gas phase diagnostics

**Figure 2:**
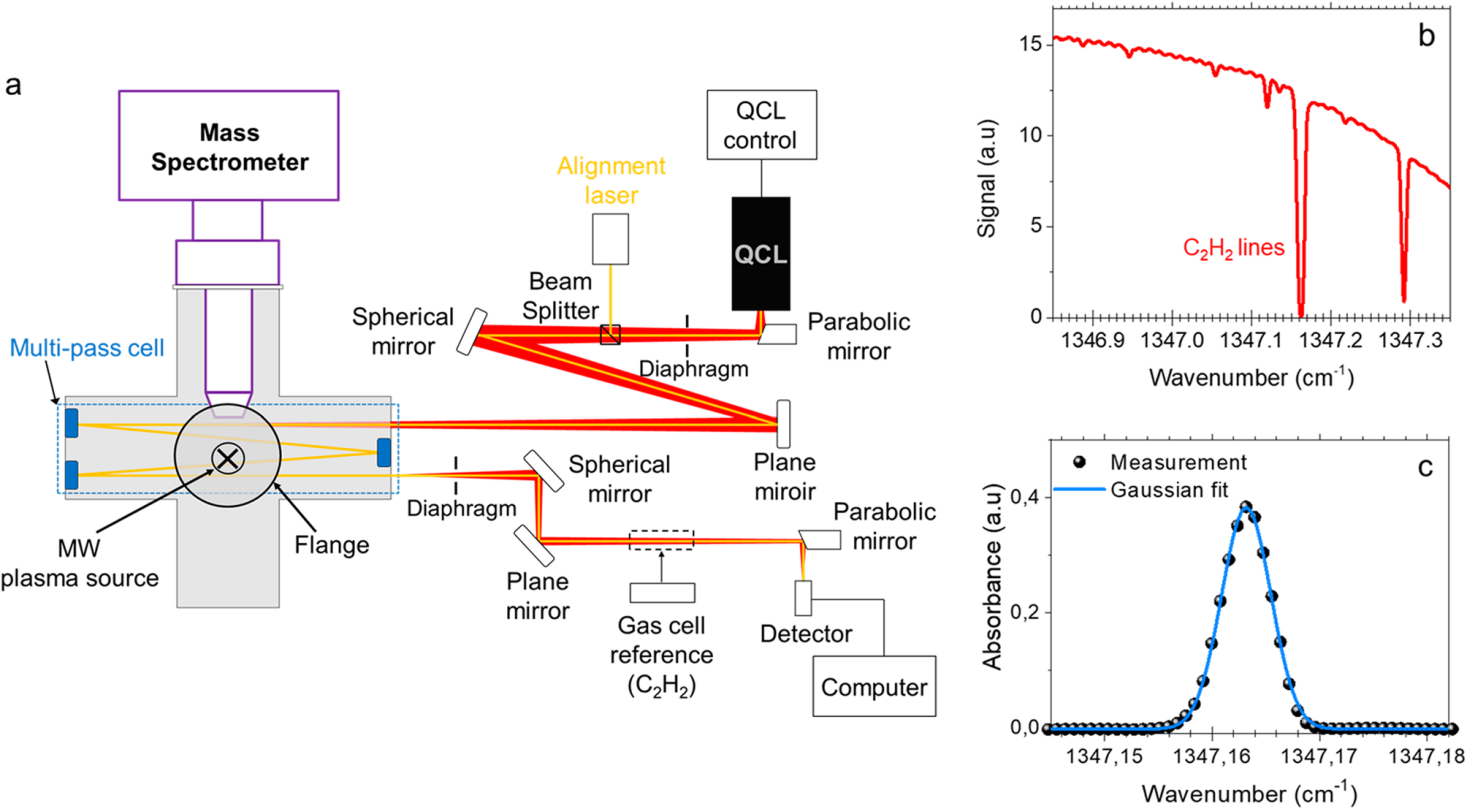
(**a**) Schematic of the diagnostics used to monitor hydrocarbon species involved in NPs formation; (**b**) a typical IR spectrum and (**c**) a Gaussian fit of C_2_H_2_ peak at 1347.163 cm^−1^.

To get insight into the chemical species that give rise to NPs nucleation, we simultaneously performed time-resolved QCLAS and MS experiments. A schematic diagram of the experimental arrangement is depicted in Fig. 2a. QCLAS experiments were performed using the Q-MACS system developed by Neoplas Control. The cw-QCL heads from Alpes Lasers SA provided a fine laser line width of about 10^−3^ cm^−1^ and a spectral range of about 10 cm^−1^ (between ~ 1340 and 1350 cm^−1^). In this spectral region, the strongest line of C_2_H_2_ at 1347.163 cm^−1^ (Fig. 2b) was monitored to determine the C_2_H_2_ density and the gas temperature *T*_*gas*_. Given our low-pressure conditions, a multi-pass White cell was integrated in the chamber to increase the absorption length up to 5 m. This enabled achieving a C_2_H_2_ detection limit as low as ~ 10^11^ cm^−3^. A reference gas cell filled with C_2_H_2_ at 3 mbar was used for both the spectral line identification and the instrumental broadening determination. Since these measurements were line of sight, an average value of both gas temperature and C_2_H_2_ density were obtained. For gas temperature measurement, the absorption line was fitted by a Gaussian function as shown in Fig. 2c. The Doppler broadening was inferred from the measured line broadening by subtracting the instrumental broadening that had been determined using the C_2_H_2_ gas reference cell at room temperature.

The gas temperature *T*_*gas*_ was then determined from the Doppler broadening *∆λ*_*D*_ using the following expression:

1 $$\Delta {\lambda }_{D}=\frac{2{\upsilon }_{0}}{c}\sqrt{2{k}_{B}{N}_{A}ln(2)\frac{{T}_{gas}}{M}}$$

where *ν*_*0*_ is the wavenumber of the spectral line transition (cm^−1^), *M* the molar mass (kg.mol^−1^), *c* the speed of light (m s^−1^), *k*_*B*_ the Boltzmann’s constant and *N*_*A*_ Avogadro’s number (mol^−1^).

The C_2_H_2_ density [C_2_H_2_] was determined using Beer-Lambert’s law:

2 $$[{C}_{2}{H}_{2}]= \frac{1}{S(T)\cdot L}{\mathop{\int }\limits_{line}}ln\left(\frac{{I}_{0}\left(0,v\right)}{I\left(L,v\right)}\right)dv$$

where *S* is the line strength of the transition (cm^−1^/(molecule⋅cm^−2^), *L* the absorption length (cm), and *I*_*0*_ and *I* the incident and transmitted laser intensities, respectively.

The gas temperature values estimated in this discharge were typically in the range of 400–700 K. We therefore assumed a constant value for the line strength that exhibits a fairly slight variation for temperature values below 1000 K^72^. We used the value of S at T = 300 K, provided by the Hitran Database^73^.

Once the density of C_2_H_2_ was determined, the following expression was used to infer acetylene conversion yield:

3 $${C}_{2}{H}_{2} conversion (\%) =\frac{{\left[{C}_{2}{H}_{2}\right]}_{0}-\left[{C}_{2}{H}_{2}\right]}{{\left[{C}_{2}{H}_{2}\right]}_{0}} {\text{x}} 100$$

where *[C*_*2*_*H*_*2*_*]*_*0*_ and *[C*_*2*_*H*_*2*_*]* are the concentration before and after the plasma ignition, respectively.

For MS measurements, we used the EQP 500 model from Hiden (RGA mode) that enables a mass detection up to m/z ratio = 500 for neutral species at a typical time-resolution of about one hundred milliseconds. A detailed description of this well-established technique can be found in^74,75^.

For OES measurement, we used a 1-m focal length Jobin–Yvon THR 1000 spectrometer equipped with a 1800 grooves.mm^−1^ grating blazed at 450 nm and a R2949 Hamamatsu photomultiplier tube. The OES experiments were carried out using an optical fiber placed in front of one of the BK7 window of the chamber in order to monitor the temporal evolution of a single Argon line.

### In-situ and ex-situ nanoparticles diagnostics

**Figure 3:**
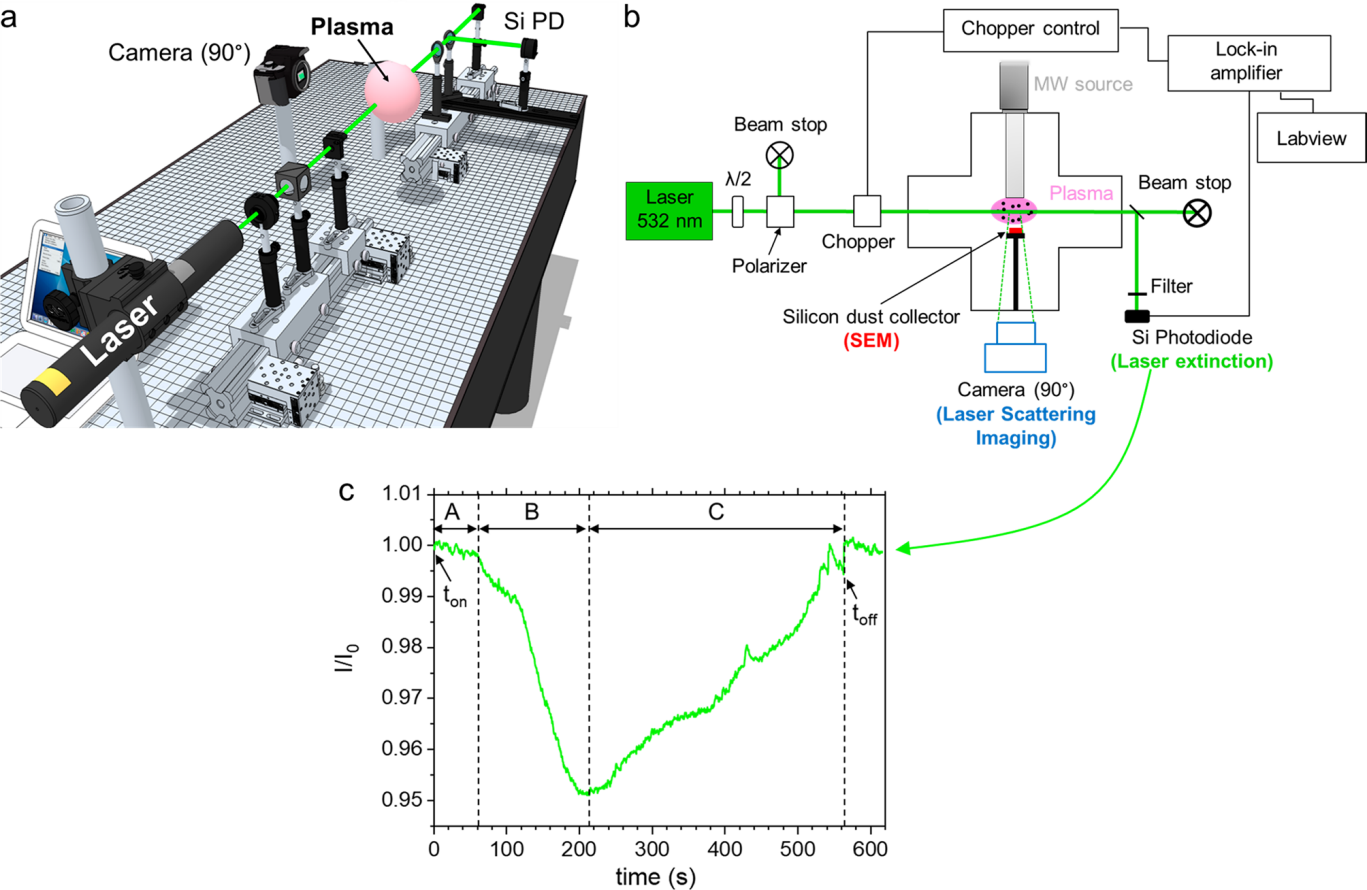
3D-view (**a**) and schematic side-view (**b**) of laser extinction/scattering experiments and (**c**) Time-resolved extinction signal recorded during dusty plasma experiment. The times at which plasma was switched on and off are indicated by the arrows. The extinction signal shows three typical regions: A = a slight decrease of the transmitted signal revealing NPs formation onset, few tens of seconds after the plasma ignition; B = a strong decrease of the transmitted signal revealing a large increase of the density and/or the size of NPs; C = an increase of the signal revealing a gradual disappearance of NPs from the laser beam path.

Figure 3 shows (a) a 3D and (b) a schematic side-view of the time-resolved in situ laser scattering and extinction experiments used to analyze the NPs cloud dynamic and growth kinetics, respectively. Both experiments were performed using a 532 nm cw-laser with a maximal output power of 1W. As far as laser extinction experiments are concerned, the power of the incident laser was modulated using an optical system composed of a half wave-plate and a polarizer. After crossing the plasma, the laser beam was split and only a small fraction was sent to a Si photodiode detector equipped with a 532 nm filter. A mechanical chopper (f = 2 kHz) connected to a lock-in amplifier was used to maximize the signal-to-noise ratio. The time-resolved intensity ratio *I/I*_*0*_ was used to monitor the NPs growth kinetic and to estimate the NPs density *n*_*p*_ along the line-of-sight laser path.

The NPs size range found under our operating condition is typically 30–180 nm, which is much smaller than the laser wavelength. As a result, the size parameter α = 2πr_p_/λ_laser_ is smaller than 1 and the particle density, *n*_*p*_, was estimated from the measured *I/I*_*0*_ ratio using Rayleigh theory^48^:

4 $$\frac{I\left(t\right)}{{I}_{0}}=exp\left[-\pi { n}_{p}\left(t\right){ C}_{ext}\left(t\right)\left({{r}_{p}\left(t\right)}^{2}\right)L\left(t\right)\right]$$

where *r*_*p*_ is the NPs radius, *L* the absorption length and *C*_*ext*_ the extinction coefficient which is the sum of the scattering and absorption coefficients *C*_*scat*_ and *C*_*abs*_ given by:

5 $${C}_{scat}=\frac{8}{3}{\alpha }^{4}{\left|\frac{{m}^{2}-1}{m+2}\right|}^{2}; {C}_{abs}=4\alpha Im{\left|\frac{{m}^{2}-1}{m+2}\right|}^{2}$$

where $\alpha =(2\pi {r}_{p}/{\lambda }_{laser})$ is the size parameter and *m* is the complex refractive index of the NPs.

To estimate the NPs density, the value of *m*, *r*_*p*_, and *L* have been respectively estimated as follows: (i) for the complex index *m* at *λ* = 532 nm, we used the values published in the literature for amorphous hydrogenated carbons (a-C:H); (ii) for *r*_*p*_, we used the mean value extracted from SEM images; (iii) for *L*, we used the lower and upper limits, corresponding to the plasma diameter, *i.e.*, absorption length of 10 cm, and the whole reactor diameter, *i.e.*, absorption length of 25 cm. Figure 3c shows a typical extinction signal from which the value of *I/I*_*0*_ was inferred for the determination of *n*_*p*_. The interpretation of this extinction signal is detailed in the caption. When it comes to the study of NPs transport and NPs cloud dynamics, we used laser light scattering imaging. For this purpose, the laser light scattered by the NPs cloud was recorded using a 33 ms time-resolution camera that was positioned perpendicularly to the laser beam. This diagnostic was used under both discharge and post-discharge conditions. When an overall view of the NPs cloud was required, the scattering experiments were carried out using a laser sheet generated from the laser beam by a cylindrical lens system. The NPs size and density were also evaluated by Scanning Electron Microscopy (SEM) using a Zeiss Supra 40 VP system equipped with a Field Electron Gun (FEG–SEM) at an acceleration voltage of 5 kV. For this purpose, Silicon substrates (1 × 1 cm) placed in front of the MW plasma source, were used to collect the NPs. The size distributions of the NPs populations collected on the silicon wafers were inferred from SEM micrographs using Image J software. The NPs size distributions were determined by counting the particles numbers for the different size ranges. They were curve-fitted by one or a combination of Gaussian distributions. Each Gaussian distribution characterizes a particle population with a specific value of the mean diameter. The relative error on NPs size was evaluated to 10%.

## Results and discussion

**Figure 4:**
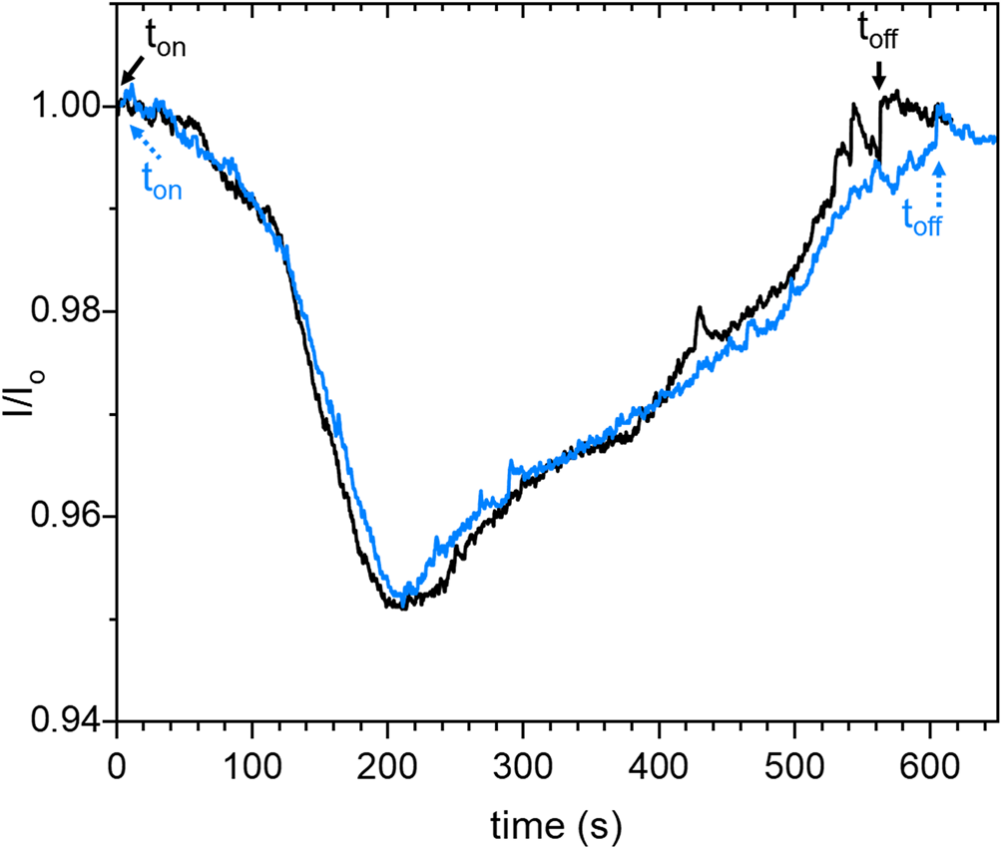
Laser extinction signals of two experiments conducted under the same operating conditions: *P* = 50 Pa/Ar:C_2_H_2_ (50:50)/Q = 3 sccm/P_MW_ = 180 W. The plasma is switched on/off at different time for both experiments. The extinction experiment is performed with the laser beam crossing the plasma volume.

As mentioned earlier, the optimal conditions that ensure NPs production were found for an equimolar mixture of Argon and acetylene Ar:C_2_H_2_ (50:50), a plasma power P_MW_ of 180 W, a pressure *P* of 50 Pa, and a total flowrate *Q* of 3 sccm. Under these conditions, we obtained a quite reasonable reproducibility in the NPs growth kinetic as shown in the extinction signals obtained for two separate experiments conducted under the same conditions during ~ 600 s plasma duration (see Fig. 4). Therefore, this dusty plasma condition is the one used in the present paper.

### Nanoparticles growth kinetic

**Figure 5:**
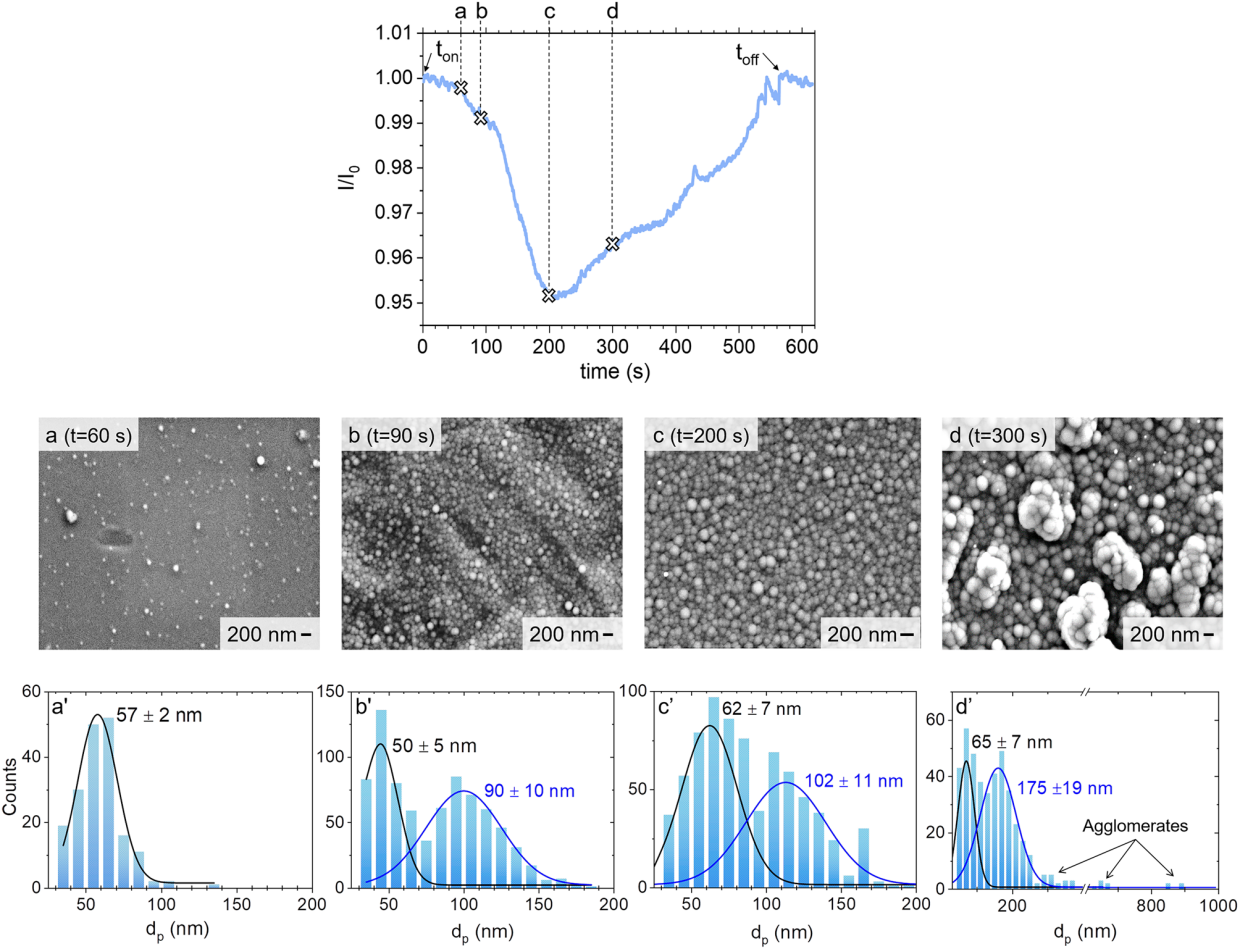
SEM images (**a**–**d**) and corresponding size distributions (**a’**–**d’**) obtained for plasma duration of 60 s, 90 s, 200 s and 300 s. Operating conditions: *P* = 50 Pa/Ar:C_2_H_2_ (50:50)/Q = 3 sccm/P_MW_ = 180 W. The laser extinction signal of figure has been added here to show the extinction signal corresponding to each SEM/size distribution. The extinction experiment is performed with the laser path crossing the plasma volume.

The time-evolution of the NPs size and density were analyzed using SEM micrographs and laser extinction signals. As far as SEM analyses are concerned, the collector was introduced in the chamber before the plasma was switched on and then exposed to the plasma for a given time-period. Once the plasma was switched off, the substrates were extracted from the chamber and then analyzed by SEM. This procedure was performed at four different plasma durations (60, 90, 200 and 300 s) as indicated in Fig. 5 that shows the SEM images and the corresponding size distributions.

#### After 60 s plasma duration

Figure 5a/a’, a small amount of spherical NPs was deposited on the silicon substrate. The size distribution can be well represented by a single Gaussian distribution with a mean diameter *d*_*p*_ of approximately 57 nm.

#### After 90 s plasma duration

Figure 5b/b’, a large increase of NPs density is observed, which is likely due to an enhanced nucleation in the gas phase. In addition, the Gaussian curve-fit of the size frequencies shows the existence of two populations with mean diameter values of approximately 50 and 90 nm, respectively. The 90 nm-diameter population would very likely result from a further growth of the particles that have been already observed after 60 s plasma duration, while the smaller 50 nm mean-diameter population would likely correspond to NPs that nucleated and grew between 60 and 90 s. This evolution in both NPs density and size between 60 and 90 s is well correlated to the faster drop experienced by the laser transmission signal after t ~ 60 s, *i.e.*, from point **a** to point **b** in the extinction signal of Fig. 5, which can be attributed to an enhanced increase of NPs density and/or size.

#### At 200 s plasma duration

**Figure 6:**
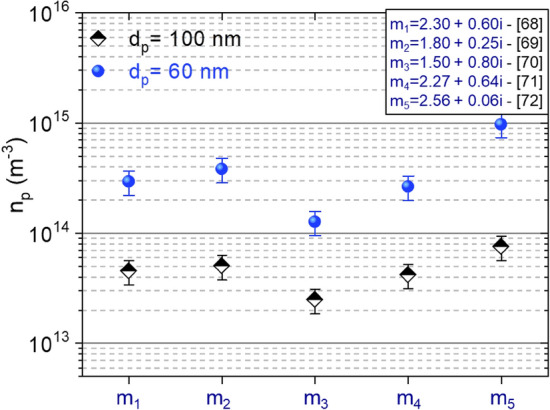
NPs density as a function of the NPs size estimated from SEM, the uncertainty due to the absorption length is represented by the error bars and the complex index m_1-5_ are taken from the literature, m_1_:^76^ ; m_2_:^77^; m_3_:^78^; m_4_:^79^; m_5_:^80^.

Figure 5c/c’, we still observe two populations with average diameters of approximately 62 and 102 nm. These correspond to the growth, through surface sticking of gas-phase species, of the two populations that were already present at 90 s. However, we observe that the growth rate of the two populations from 90 to 200 s is quite low (~ 15%) whereas the transmission signal keeps experiencing a large drop during the same period. This signal drop corresponds therefore necessarily to an increase in the NPs density. We estimate the NPs density values from the extinction signal measured at this time period using Eqs. (4–5) on the basis of the assumptions discussed in Sect. “In-situ and ex-situ nanoparticles diagnostics” using different values of the complex index. The results are depicted Fig. 6 that shows that the NPs density is in the range of 4 × 10^13^ to ~ 4 × 10^14^ m^−3^. Note that the major source for the uncertainty on the density value are NPs mean diameter and of the complex indexes, the absorption length having a fairly limited impact.

#### At 300 s plasma duration

Figure 5d/d’, we observe two populations of NPs, respectively centered at 65 nm and 175 nm, along with quasi-micrometric agglomerates, indicated by the arrows. Small particles with diameter lower than 50 nm are also still observed.

In summary, both laser extinction experiments performed in the central plasma region and SEM characterizations clearly show the formation of NPs that grow continuously in the plasma. Once the NPs density reaches a value around 10^14^ m^−3^ (Fig. 6) and the NP’s size a value of the order of 100–300 nm, they tend to agglomerate and the extinction signal shows a significant decrease of the particle density in the plasma region.

### Nanoparticles transport dynamic

#### Characteristics of the nanoparticles cloud

**Figure 7:**
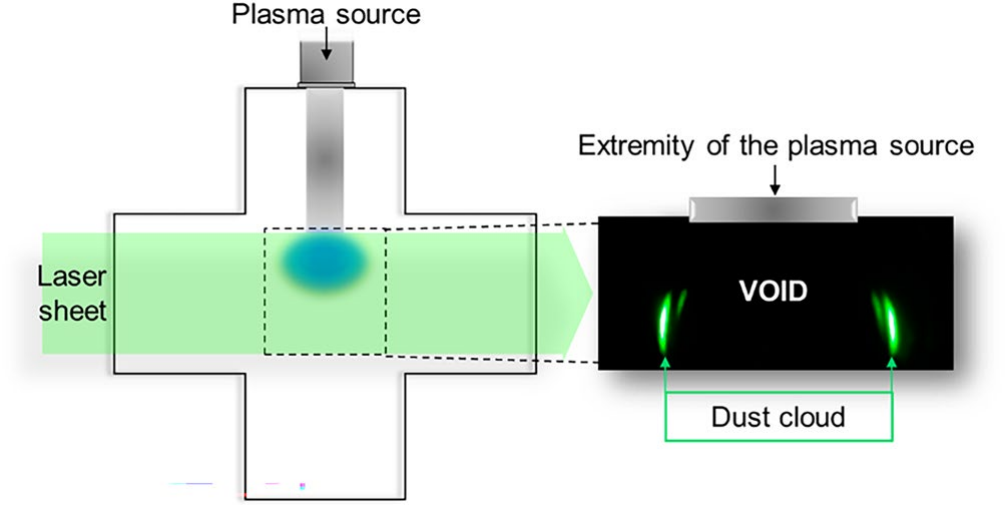
Laser scattering imaging set-up. The inset shows the toroidal NPs cloud with the void region when capturing an image with a camera at 90°.

To obtain the topological characteristics of the NPs cloud, we used laser light scattering imaging. We illuminated the NPs cloud by a laser sheet and then recorded the laser light scattering images at 90° right after the plasma extinction so as to get rid of the plasma emission. An example of such images is given in the insert of Fig. 7 that shows a cross-sectional view of the NPs cloud and clearly reveals the existence of a void region. This image shows the toroidal shape of the cross section of the NPs cloud by the laser beam and reveals that the void region corresponds approximately to the plasma emissive volume. It appears therefore that the decrease of the particle population density in the plasma region evidenced by the extinction experiment discussed in the previous section is induced by the formation of the void region.

It is noteworthy that for all the experiments performed in this work, we observed that the major fraction of the NPs cloud was located outside the plasma emissive volume when the plasma duration exceeds ~ 100 s.

**Figure 8:**
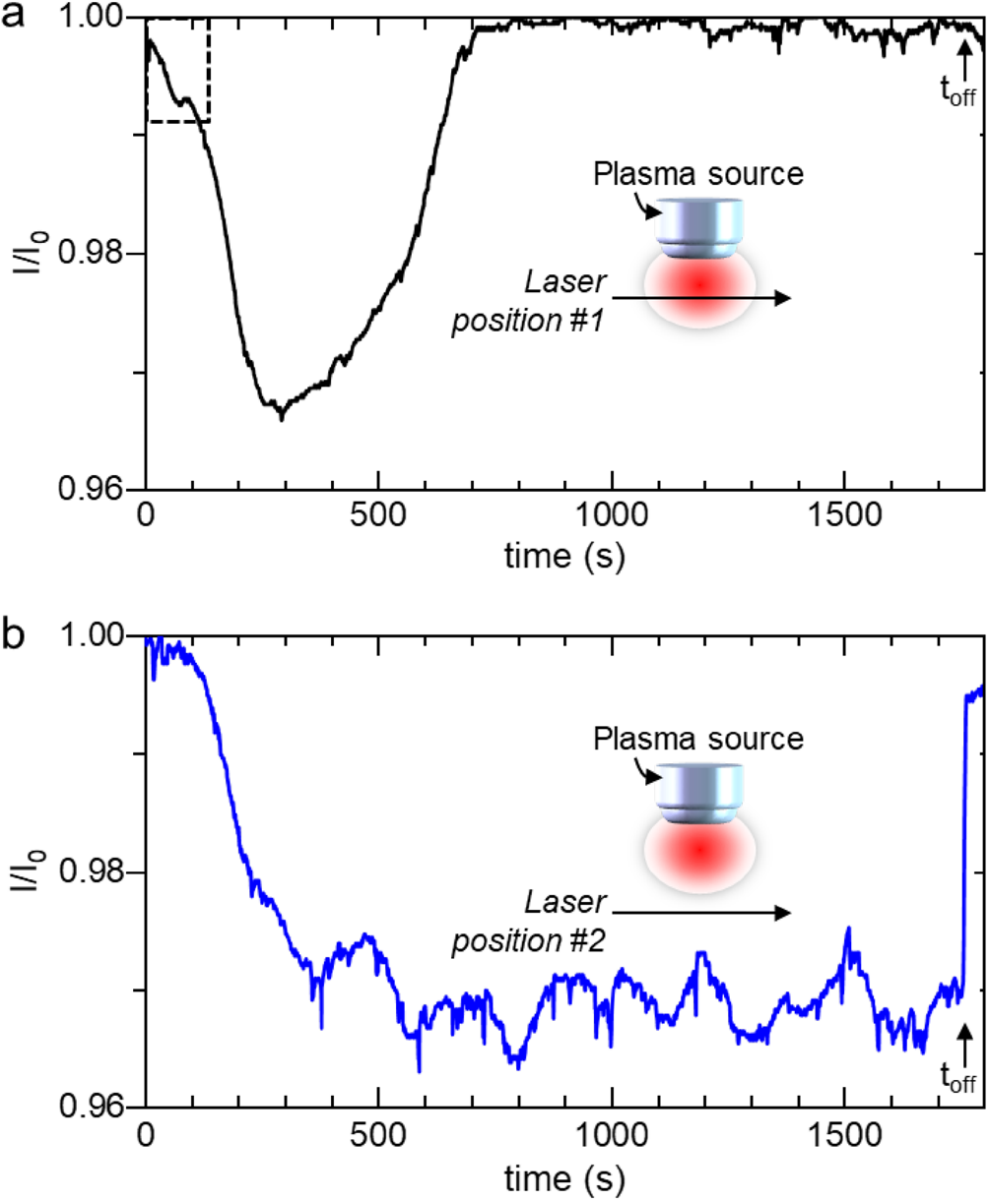
Spatially resolved laser extinction signals when (**a**) the laser beam crosses the center of the plasma volume and (**b**) propagates outside the plasma. Plasma was ignited at t = 0 s.

To further characterize the NPs cloud dynamics, time-resolved line-of-sight laser extinction experiments were carried out in two configurations for longer plasmas durations, i.e., 1800s. In the first configuration, the laser beam crosses the center of the plasma volume (Fig. 8a) while in the second one the laser propagates outside the plasma (Fig. 8b).

The extinction signal obtained when the laser beam propagates across the plasma volume (Fig. 8a) shows that the particle cloud develops inside the plasma during 300 s, i.e., the transmission signal decreases, then the particle are transported outside the plasma volume thus resulting in the formation of the void that develops between 300 and 700 s. Above 700 s plasma-duration, the extinction signal is almost zero, indicating that the plasma region does not contain solid particles.

When the laser propagates outside the plasma volume (Fig. 8b), the signal shows a very different evolution. After a first decrease that takes place during 300–400 s and which indicates that the dust particles populate the post discharge region at the periphery of the plasma volume, the transmission signal reaches a permanent regime with quasi-periodic time-variations. It appears therefore that, after a first development phase, the volume fraction of the dust particles remains significant and almost constant when the plasma is on. Once the plasma was switched off, the extinction signal sharply increased, which indicates that NPs are rapidly expelled from the volume surrounding the plasma at the extinction of this later.

#### On the origin of the void and nanoparticles agglomeration

Further insights in NPs dynamic may be inferred by combining laser light diffusion experiment and a simple numerical model. We mainly focused on the post-discharge phase, right after the plasma extinction, in order to cancel the interference between the plasma emission and the diffused signal on one hand, and to keep the momentum balance that governs the NPs dynamics relatively simple.

**Figure 9:**
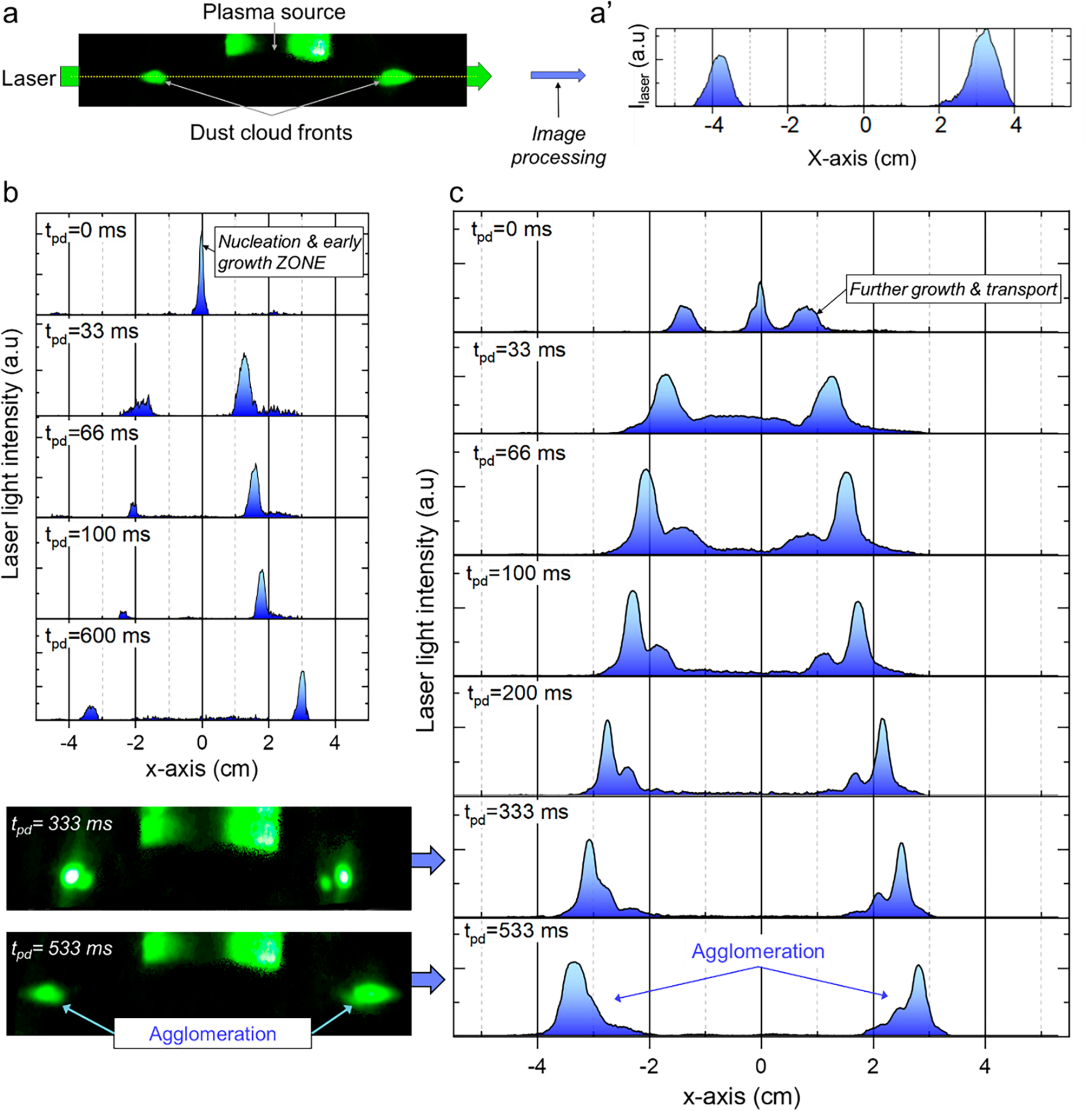
(**a**) Cross section image showing the laser light scattering of the NPs cloud (**a**). Note that the plasma source also appears green due to laser reflections and (**a’**) the corresponding diffused laser light intensity profile; (**b**)-(**c**): time-evolution of the NPs cloud cross section during the post-discharge phase of a plasma that has been sustained during 60 and 100 s, respectively.

The NPs cloud transport was monitored in the post-discharge with a time-step resolution of 33 ms. The cross section of the laser light scattered by the NPs cloud (Fig. 9a) was processed (Fig. 9a’) to monitor the dynamic of the NPs cloud front. We first performed experiments during the post-discharge phase of a plasma that was sustained during only 60 s, a period of time during which only a single generation of NPs is produced as shown previously by SEM/laser extinction results. The time-evolution of the NPs cloud cross-section in the post-discharge (*t*_*pd*_ refers to the post-discharge duration) is shown in Fig. 9b. Then, in a second experiment (Fig. 9c), we captured the dynamics of the NPs cloud cross-section during the post-discharge phase that takes place after 100 s of plasma duration.

In Fig. 9a, we observe the presence of a single peak at (x = 0/*t*_*pd*_ = 0 ms), representing the cloud of a first generation of NPs. This cloud is confined in the center of the plasma. Once the plasma is switched off, for *t*_*pd*_ > 0 ms, the NPs cloud was pushed out radially toward the reactor wall at an approximately constant velocity of about 0.5 m s^−1^. This behavior may be easily interpreted as follows. When the plasma is on, the positive plasma potential tends to push the negatively charged particles to the center of the plasma, while other forces such as ion drag, thermophoresis or diffusion tend to push the particles outside the plasma volume. It appears therefore that for a population with 50 nm average diameter the equilibrium between these forces results in a particle cloud positioned at the center of the plasma. Once the plasma is switched off, the plasma recombines and the dust particles neutralize rapidly. As a result, they are no more subject to the electrostatic or ion drag forces. The observed particle transport can be only induced by diffusion or thermophoresis. However, diffusion-dominated transport cannot lead to a toroidal-like particle cloud such as the one obtained in Fig. 9a, but, rather, to a wider spatial distribution with the same maximum position. It appears therefore particle transport during the early post discharge phase is very likely governed by the thermophoresis force.

For longer plasma duration, *i.e.*, 100 s (Fig. 9b), two different particle populations are observed. A first one at the center of the plasma represents the cloud of the particles that nucleate and experience an early grow in the central region of the plasma .This first population shows the same equilibrium position as the one observed after 60 s plasma duration and should therefore involve particles with size of approximately 50 nm or below. Those observed at (x =  +/− 1 cm/*t*_*pd*_ = 0 ms) correspond to NPs for which the forces acting against electrostatic trapping when the plasma is on, i.e., ion drag, thermophoresis and diffusion, are larger than for the 50 nm diameter population. This means that the particles of this second population have a larger diameter. This second population corresponds therefore to the second distribution mode, with an average diameter of approximately 100 nm, observed on the SEM picture corresponding to 100 s plasma duration in Fig. 5b,b’.

Figure 9a,b show that a first population of particles nucleates and experiences a first growth phase up to a size of the order of 50 nm at the center of the plasma. When these particles experience a further growth the balance between electrostatic and thermophoresis forces evolves and the equilibrium position of this first particle cloud shift away from the plasma center. Subsequently, the particle cloud adopts a toroidal shape and the central region of the plasma is subject to dust particle depletion before a second population corresponding to the central cloud in Fig. 9b nucleates and grows. This scenario is confirmed by laser extinction measurements. As a matter of fact, one can notice that when the laser crosses the plasma (Fig. 8a), the extinction signal exhibits a short kink between ~ 20 and 100 s (evidenced by a dotted square box). The decrease of the extinction signal before the occurrence of the kink corresponds to the formation of a first NPs generation. The occurrence of the kink tends to indicate that the particles of this first generation are expelled from the plasma volume while nucleation and growth keep on taking place in the plasma phase, which results in the strong decrease of the signal immediately observed after the kink.

For longer post-discharge duration (Fig. 9c/t_pd_ = 333 and 533 ms), the NPs clouds tend to slow down, merge and end up in a single cloud.

#### Momentum balance for nanoparticles: model

To identify the major forces that drive NPs transport in the investigated discharge, we have to perform a force balance taking into account all the potential forces that act on the NPs in the plasma. We can distinguish two groups of forces:

(i) Those depending on the plasma parameters and the charging behavior of NPs, *i.e.*, electrostatic *F*_*E*_ and ion drag force *F*_*i*_.
(ii) Those that do not depend on these parameters and that are still acting during the post-discharge phase, *i.e.*, the thermophoresis force *F*_*th*_, diffusion, neutral drag force *F*_*N*_ and gravity force* F*_*g*_*.*

We consider the NPs transport only during the post-discharge phase so as we can get rid of electrostatic and ion drag forces and use laser scattering experiments (cf. Figure 9) that were performed after the plasma extinction.

As a matter of fact, although particles with residual charges may still exist, the major part of the particles recombines very rapidly once the plasma is switched off. Additionally, the electric field is very weak during the post-discharge phase of the considered electrodeless microwave plasmas. As a result, the electric field driven forces, and more particularly electrostatic and ion drag forces, become very weak and can be disregarded in the momentum balance equation during the post-discharge phase^54^. Further, as discussed previously, the evolution of the particle cloud obtained after 60 s plasma duration during the post-discharge shows that diffusion can be also disregarded.

On the opposite, regarding the low thermal diffusivity in our operating conditions, thermal gradients are still effective after plasma extinction even for long post-discharge durations (> > 1 s). Subsequently, the particle cloud transport can be only governed by neutral drag, thermophoresis and gravity during the post-discharge phase. Considering the size of the particles involved here, the gravity force F_G_(α d^3^) can be neglected. It appears therefore that the transport of NPs during the post-discharge phase will be likely dominated by thermophoresis force *F*_*th*_ related to Soret effect and the neutral drag force *F*_*N*_.

The above analysis enables a straightforward evaluation of the thermophoresis and neutral drag forces by comparing the particle cloud dynamics determined experimentally from laser scattering experiments and the particle dynamic calculated using a two-dimensional momentum balance equations during the post-discharge. This equation can be written:6

**Figure 10:**
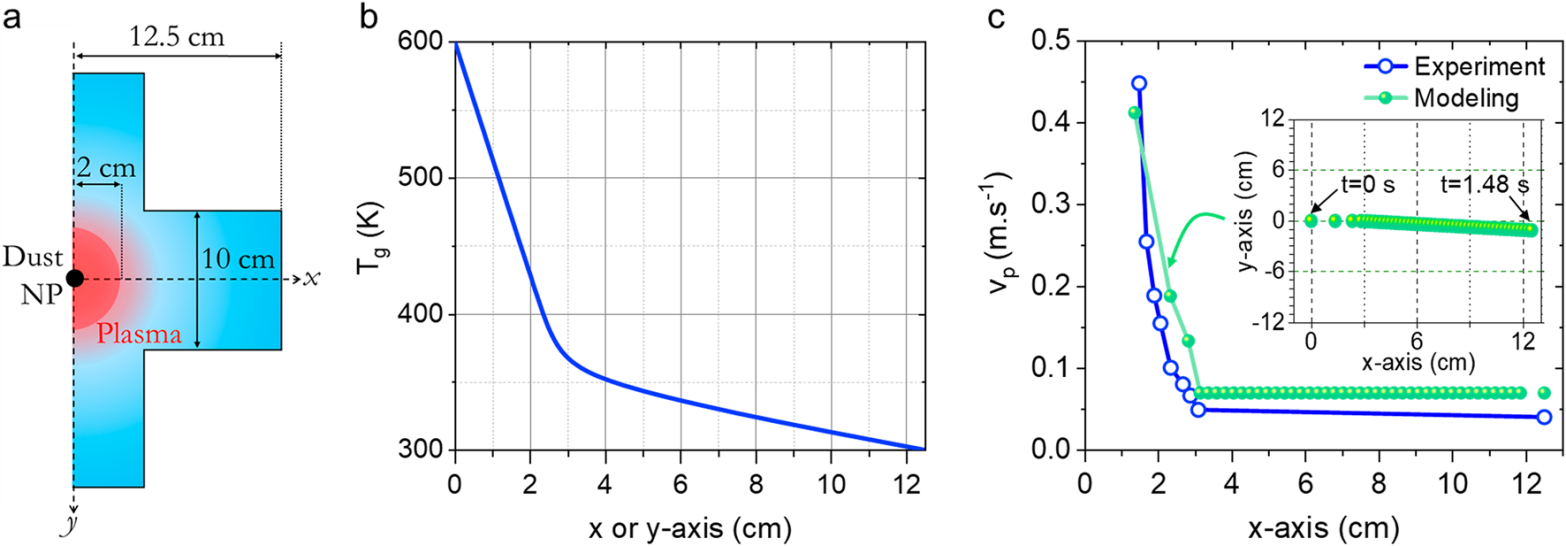
(**a**) The schematic view of the geometry used for the model; (**b**) temperature profile along the radius of the chamber and (**c**) spatial particle velocity (experiment *vs* modeling): the inset shows the position of the cloud as a function of time in the plasma chamber from which the modeling velocity has been extracted.

In Eq. (6), the thermophoresis force expression corresponds to the free molecular regime^82^ while the expression from Epstein was used for neutral drag force^83^. *r*_*p*_ is the NPs radius; *α*_*T*_ the thermal conductivity of the gas; $\overrightarrow{\nabla }T$ the thermal gradient; *T*_*n*_ the neutral temperature; *m*_*n*_ the neutral mass; *n*_*n*_ is the neutral density, *v*_*p*_ the NPs speed, *v*_*n*_ the neutral speed and α a coefficient, corresponding to the reflection/diffusion of the gas atom on NPs is equal to 1. All the input parameters for the calculation are given in Table 1. The determination of *F*_*th*_ requires the spatial profile of *T*_*n*_ which also gives $\overrightarrow{\nabla }T$. This was inferred by adjusting the temperature profile so as to achieve the best curve-fit between the spatial variation of the particle velocity (and the subsequent acceleration) calculated by Eq. (6) and measured by laser scattering. In this curve-fitting procedure, the plasma is axisymmetric; the adopted geometry is represented in Fig. 10a. As inputs, we consider a single spherical NP of a radius *r*_*p*_ estimated from SEM images.

**Table 1:** Value of the parameters appearing in Eq. (6).

Symbol	Value
r_p_	50 nm
Α	1
T_n_ (mean temperature)	450 K
T_wall_	300 K
m_n_	0.5 m_Ar_ + 0.5 m_C2H2_
n_n_	P/k_b_T_n_
Ρ	ρ_carbon_ = 2000 kg.m^−3^
v_n_	0.01 m s^−1^ (3 sccm at 50 Pa) and S = 78 cm^2^

Since, as shown earlier, the NPs are confined in the plasma when their size is smaller than ~ 100 nm, we assumed an initial velocity v_0_ = 0 m s^−1^. Based on QCLAS measurement, we set the temperature in the center of the plasma at a value of 600 K while the wall temperature *T*_*wall*_ was set to 300 K. The temperature profile along the radius of the chamber obtained using the above-mentioned procedure is shown in Fig. 10b. The output of this model provides the NPs velocity profile from the reactor center to the wall of the reactor. The simulation is stopped when the NP reaches the wall. Typically, right after the plasma extinction, the NP is ejected to the wall with a velocity close to 0.4 m s^−1^.

Figure 10c compares the space-variation of particle velocity measured by laser scattering and its best curve-fit by Eq. (6). These show a fairly good agreement indicating that the thermophoresis is the dominant force during the post-discharge phase. For a NP size of 100 nm, the value of *F*_*th*_ would be approximately 6.10^−15^ N. In comparison, a rough estimate of *F*_*E*_ and *F*_*i*_ during the discharge phase would give values of ~ 5.10^−14^ and 5.10^−15^ N respectively, if one considers the following conditions: a fairly low electric field in our condition, *i.e.* less than 5 V/cm, *T*_*e*_ ~ 3 eV, *n*_*e*_ ~ 5.10^9^ cm^−3^. This implies that for particles of 100 nm, the thermophoresis would be of the same order of magnitude as the electrical forces. However, as long as their size is smaller than ~ 100 nm, the NPs grow within the plasma, where they are confined by the electrostatic force. When they reach a critical size of about 90 nm (estimated from the results shown in Fig. 9), they are gradually pushed away from the plasma by thermophoresis toward the afterglow region where they experience further growth. This effect is the driver for the void formation observed on the extinction signals outside the plasma volume. Outside the void region, in the post-discharge at the periphery of the plasma, clouds merging process involving successive trains of NPs transported from the bulk of the plasma takes place. In this region, the volume fraction of the dust particles remains important as long as the plasma is on. As a result, particle coagulation and agglomeration take place and ends up with the sedimentation of agglomerated particles onto the substrate as observed in Fig. 5d. This series of phenomena consisting of (i) nucleation and early growth at the center of the plasma, (ii) transport to the post-discharge at the periphery of the plasma and (iii) coagulation/agglomeration and sedimentation, results in the long time-scale periodic expansion/contraction cycles observed on the extinction signal of Fig. 8b. These effects are specific to the investigated microwave discharge configuration that makes use of a localized plasma source that yields a significant thermal gradient which is responsible for the void formation. This is different from parallel plate RF discharges, which are characterized by relatively low thermal gradients and where the dust particle transport is usually governed by the balance between the electrostatic forces and the ion drag forces. This usually results in a particle cloud located in the vicinity of the sheath edges when NPs diameter becomes larger than few tens of nm.

### Nanoparticles nucleation process

In the previous sections, we have demonstrated the growth of NPs at density- and diameter-values in the range of 10^13^–10^14^ m^−3^ and approximately 100 nm, respectively. In addition, we have observed that the NPs growth kinetic rapidly evolves resulting in the enhancement of NPs size and density for plasma durations exceeding 100 s. This would result in a significant contribution of the NPs in the charge and species balance of the plasma. Consequently, the electron density and the plasma composition are very likely to be affected by the presence of the NPs. This effect is investigated in this section by monitoring the emission signal of Ar line that reflects the electron dynamics, and the QCLAS/mass spectrometry signals for a variety of hydrocarbon species involved in the chemistry of C_2_H_2_ dissociation and NPs nucleation. Conversely, the investigation of electron dynamics and plasma composition also helps in shedding light on the particle formation kinetics not only during the growth phases, as inferred from laser diagnostics, but also during the molecular clustering phase that leads to nucleation.

#### Electron kinetic during NPs growth

In dusty plasmas, the electrons are substantially affected by the presence of NPs via attachment reactions. This especially takes place at large NPs density. In typical non-equilibrium discharges, *i.e.*, *n*_*e*_ = 10^9^–10^11^ cm^3^ and *T*_*e*_ = 2–5 eV, the particles acquire their ‘equilibrium’ OML charges over time scales lower than 1 ms^84^. Therefore, for timescales far above milliseconds, the particle charge may be considered as quasi-stationary, i.e., the electrons react quasi-instantaneously to any change in the particle size-distribution. Therefore, the time-variations of the particle population in the discharge may be monitored by following the electron dynamics, as long as the investigated timescales are above the charging time-scale. Therefore, we used time-resolved OES to follow an Argon emission line (738.4 nm) that indirectly probes the electron dynamic.

**Figure 11:**
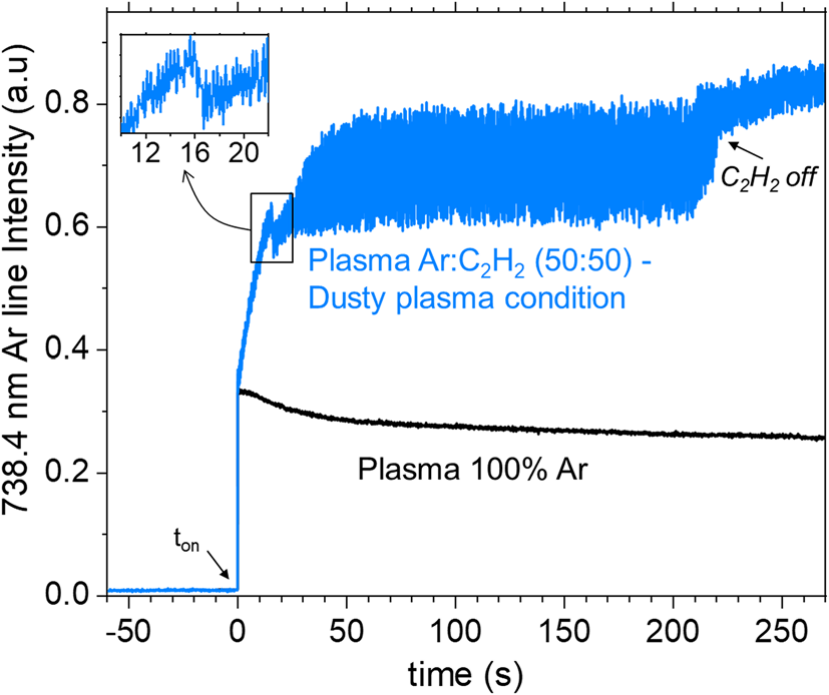
Time-resolved Ar emission line intensity for dusty plasma condition, *i.e*., Ar:C_2_H_2_ (50:50) and dust-free plasma conditions, *i.e.,* 100% Ar.

Figure 11 shows the time-evolution of the Ar line intensity for standard dusty plasma condition, *i.e.*, Ar/C_2_H_2_ (50:50). For comparison purpose, we show the emission intensity obtained in 100% pristine Argon plasma. As far as dusty plasma conditions are concerned, C_2_H_2_ flow was turned off at t ~ 215 s to further emphasize the change in the argon line intensity when acetylene is no longer present in the discharge. In Fig. 11, we observe that for 100% argon plasma condition, the Ar line intensity reaches a quasi-steady value for duration above 50 s while in dusty plasma conditions, it shows strong amplitude fluctuations, *i.e.*, almost 30% of the signal amplitude, during the whole discharge phase. When C_2_H_2_ flow is turned off, the intensity is affected in two ways:

(i) The average intensity increases, which may be attributed to an increase in the electron density due to the reduction of the attachment kinetics and/or of the electron losses at the surface of NPs. It is however very likely that the later effect is the most significant since its kinetics is much faster than the attachment kinetics for the particle size and density values estimated in the investigated discharge^84^;
(ii) The fluctuation amplitude, although still present, decreases substantially. This demonstrates that a significant fraction of these fluctuations were indeed related to the presence of NPs in acetylene containing plasmas.

Another interesting point is that the line emission’s fluctuation builds-up between 15 and 60 s, which is consistent with the time required for the formation of NPs at a significant density level to perturb the plasma. Before this strong fluctuation regime, we observe a rapid increase of the Ar line intensity during approximately 15 s after the plasma ignition. This is likely due to the fact that at the early stage of the plasma the size and density of NPs are too small to perturb the space charge field and the electron density and temperature. Once the particles reach a significant size/density, they are more prone to affect significantly the space charge equilibrium and the electron kinetics, which ends up with larger fluctuations. This supports again the idea of a close relation between the presence of NPs and the fluctuation of Ar emission line intensity. In fact, even though the acetylene flow is turned off, NPs are still present in the volume as shown earlier.

#### Hydrocarbon precursors kinetic

**Figure 12:**
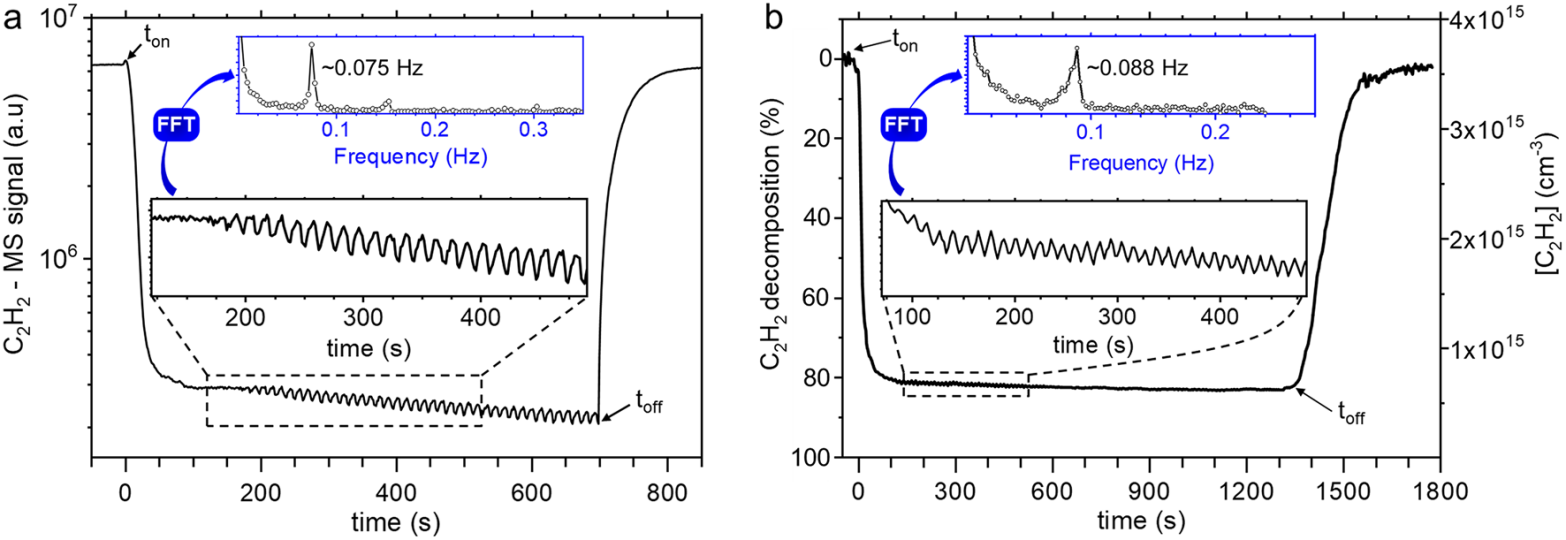
(**a**) MS signal of C_2_H_2_ and (**b**) C_2_H_2_ concentration and decomposition degree, as a function of time_._ Measurements have been done under identical conditions: *P* = 50 Pa/Ar:C_2_H_2_ (50:50)/Q = 3 sccm/P_MW_ = 180 W.

Nanoparticle formation starts with the dissociation of C_2_H_2_ molecule through electron impact, which results in the formation of neutral and charged hydrocarbon products, some of which undergo molecular growth processes that end up with the nucleation of solid NPs. In order to gain insight in the chemistry involved during NPs formation, we monitored the kinetic of C_2_H_2_ molecule along with the neutral species resulting from its conversion. Figure 12 shows the time-resolved mass spectrometry signal of C_2_H_2_ (Fig. 12a), along with the time-variation of C_2_H_2_ density and conversion yield acquired with QCLAS (Fig. 12b) under dusty plasma conditions. In both figures, we observe a fast decrease of the signal intensity after the plasma ignition. This decrease lasts approximately 30 s which therefore corresponds to the characteristic time for C_2_H_2_ conversion. The conversion yield reaches 80% (Fig. 12b), which means that other hydrocarbon species may be present at a significant amount in the plasma. Beyond 30 s plasma duration, both MS and QCLAS acetylene signals show a very slight long-term decrease. A close look to the very slowly evolving MS and QCLAS signals clearly shows the existence of periodic oscillations. These oscillations are evidenced in the square boxes in Fig. 12a,b. They start approximately 200 s after the plasma ignition, a time period that coincides with the time at which laser extinction measurement exhibit a minimum transmission, i.e. a maximum volume fraction of NPs (see \* MERGEFORMAT Fig. 8 for example).

A Fast Fourier Transform procedure performed on both signals shows that these fluctuations exhibit frequency of the order of 10^−1^ Hz. These are very likely associated to a self-consistent dusty plasma effect that has already been observed by other authors. For example, in the case of RF dusty plasmas, Mikikian et al*.*, have attributed these instabilities to the dynamic of the void region^41^.

**Figure 13:**
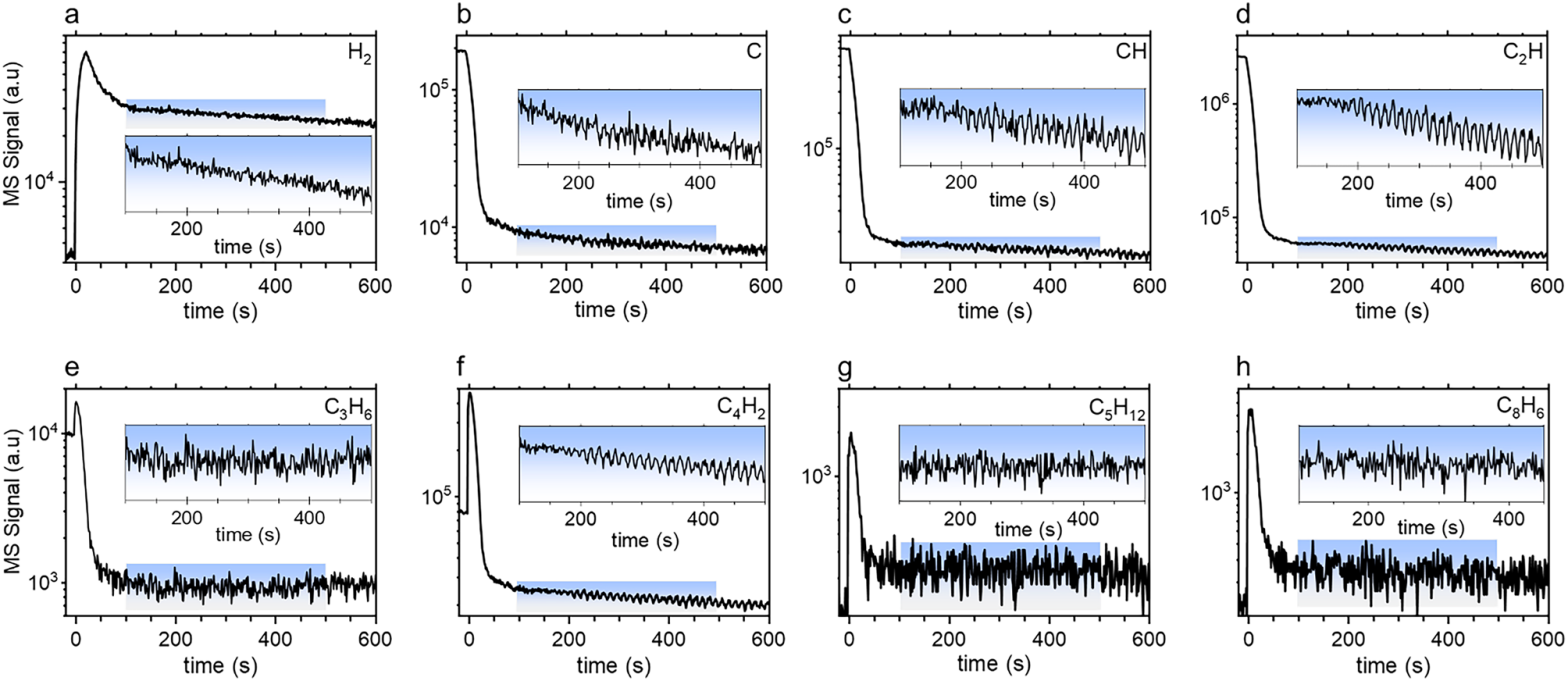
Time-resolved MS measurements of H_2_, C and several hydrocarbon species. The blue square boxes in each graphs represent the zoom region of which the graph is plotted as inset. The plasma has been switched on at t = 0 s. Operating conditions: *P* = 50 Pa/Ar:C_2_H_2_ (50:50)/Q = 3 sccm/P_MW_ = 180 W.

We also observed fluctuations on other species that originated from the dissociation of C_2_H_2_. This is shown in Fig. 13 that depicts the MS signals of H_2_ (Fig. 13a), carbon (Fig. 13b) and hydrocarbons (Fig. 13c–h). These fluctuations, start approximately 200 s after the plasma ignition as in the case of C_2_H_2_ (Fig. 12a). They are much pronounced for some species such as C, CH, C_2_H the mass signals of which are shown in Fig. 13b–d. Figure 13 also shows that the signals of these species closely follow the variation trend of C_2_H_2_ density. Also interesting is the fact that the relative decreases of the MS signals of these species are of little more than one order of magnitude, which is also consistent with the decrease in the MS signal of C_2_H_2_. In any case, these results clearly show that the characteristic time for acetylene dissociation is approximately 30 s, which is consistent with the onset time required for of NP’s production inferred from OES measurement, *i.e.*, ≈15 s. Figure 13 also shows the time-variations of some large hydrocarbon species that are likely to be involved in the molecular growth processes leading to NPs nucleation. These species behave differently compared to smaller hydrocarbon species. In particular, they experience a first strong increase during 30 s after the plasma ignition and then a decrease before reaching a permanent regime where fluctuations, similar to those observed for acetylene, can be easily observed for large hydrocarbon species that exhibit strong signals, *i.e.*, C_4_H_2_ (Fig. 13f). These results show that molecular growth of hydrocarbons in argon/acetylene plasma is a fairly fast process in agreement with what has been suggested by modeling results for RF discharges^85^. Also interesting, is the case of molecular hydrogen (Fig. 13a) that represents a ‘terminal’ species as far as acetylene conversion and molecular growth of large polyenes are concerned^85^. Despite its large MS signal, molecular hydrogen species, unlike hydrocarbons, does not show density fluctuations during the permanent regime. This would indicate that the observed density fluctuations on HC species would be a consequence of the interaction between these species and the NPs cloud, *i.e.,* surface-reaction of hydrocarbon molecules on the NPs surface.

As a matter of fact, the fairly reactive hydrocarbon species are more sensitive to surface reactions on NPs, e.g. surface sticking, than the stable molecular hydrogen. Such an interpretation is consistent with the fact that the fluctuation appears exactly when the NPs volume-fraction, as inferred from extinction measurements, reaches its maximum value. The time-variation of H_2_ signal over long time-scales (Fig. 13a), shows a first increase consistent with the early conversion of acetylene to hydrogen-poor hydrocarbon species like polyynes, *i.e.*, C_n_H_2_, reported in RF plasma studies^64,85^. Then, H_2_ density reaches a maximum value and then decreases by a little more than a factor 2 to its quasi-steady state value. The important point here is that unlike the hydrocarbon species that show between one and two order of magnitude density decrease, the H_2_ signal during the permanent regime remains fairly large and in any case comparable to the maximum value achieved in the early stage of the discharge. This means that H_2_ is continuously produced at a significant amount in the plasma, which tends to indicate a continuous production of poor-hydrogen hydrocarbon species such as polyynes. Further, the quasi-steady state MS signals of larger hydrocarbon species given here as examples, *i.e.*, C_5_H_12_ and C_8_H_6_, (Fig. 13g,h), although noisy, indicate that molecular growth keeps taking place during the permanent regime.

## Conclusion

**Figure 14:**
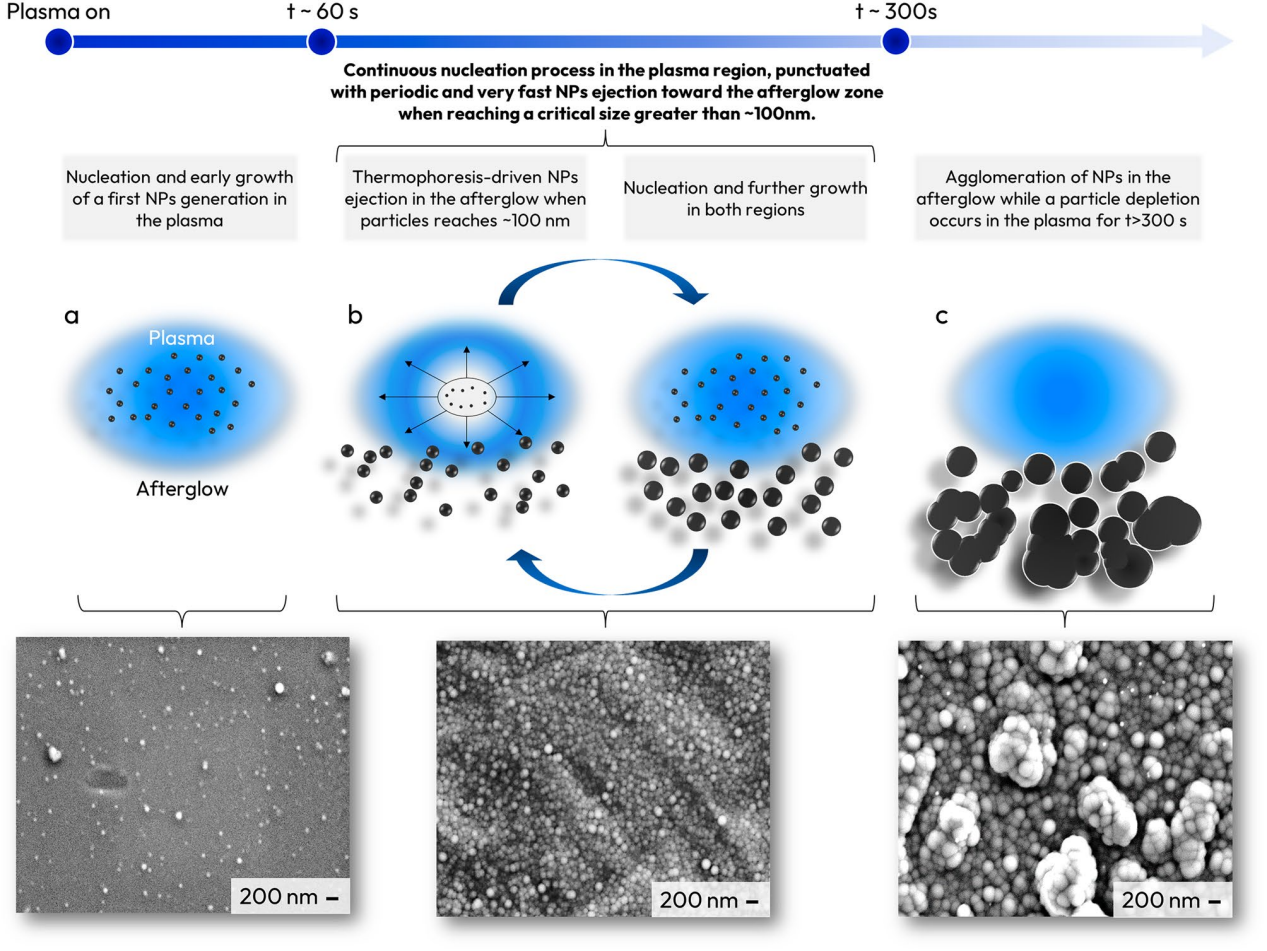
Nanoparticles formation and dynamic route in low-pressure microwave plasma.

This study, conducted with the combination of several complementary diagnostics, allowed us to understand NPs formation kinetic and NP cloud dynamic in low-pressure microwave discharges using acetylene as precursor. The corresponding mechanism is summarized in the schematic illustration presented in \* MERGEFORMAT Fig. 14 and detailed below:

- From plasma ignition to ~ 60 s: the dissociation of C_2_H_2_ molecule, that occurs during the first 30 s, mainly results in the production of hydrogen-poor hydrocarbon species, probably the polyynes C_n_H_2_, that undergo molecular growth processes which ends up with the nucleation of NPs belonging to the first generation of particles produced in the plasma. The location of the particle cloud is governed by the balance between the electrostatic force that tend to confine the particle at the center of the plasma and thermophoresis forces that tend to push the particle outside the plasma. This balance result in a particle cloud located at the center of the plasma as long as the particle size is less than 100 nm.
- Between ~ 60 s and 300 s of plasma: NPs nucleate and experience further growth through molecular sticking in the plasma region up to a size of approximately 100 nm. During this phase, the thermophoresis force (α d^2^) increases much faster than the electrostatic force (α d). As a result, the equilibrium position of the particle cloud shifts from the center of the plasma to its periphery. In particular, we showed that at approximately 100 nm, NPs formed in the plasma start to be transported by thermophoresis from the central plasma region, where nucleation takes place and small particles are trapped by electrostatic force and undergo early growth, toward the afterglow region. Indeed, the MW source used in this work produces a localized hemispherical plasma in which the gas temperature is high enough causing significant thermal gradients. This thermal gradient generates significant thermophoresis transport which leads to the formation of a void in the central plasma region. Once NPs are transported outside the central plasma region, they experience further growth in the afterglow.
- From ~ 300 s onward, particle depletion takes place in the central region of the plasma where the particle volume fraction decreases to almost zero after 600 s plasma duration. At this stage, the central region of the plasma is almost free of particles. Extinction experiments show that particles accumulate in the afterglow region at the plasma periphery and show a quasi-stationary volume fraction with a long time-scale oscillation as long as the plasma is on. The analysis of scattered light images shows that NPs are mostly, if not all, located in the afterglow while the nucleation in the plasma is strongly reduced due to the acetylene depletion after 200–300 s discharge duration. Once in the post-discharge, the charging process is no more active and the particles carry limited number of residual charges. Coagulation and agglomeration can therefore take place and lead to the 1 µm size agglomerates observed on the silicon collectors

## Data Availability

The datasets generated during and/or analysed during the current study are available from the corresponding author on reasonable request.
